# An electrically conductive metallocycle: densely packed molecular hexagons with π-stacked radicals†

**DOI:** 10.1039/d2sc00447j

**Published:** 2022-04-01

**Authors:** Mengxing Cui, Ryuichi Murase, Yongbing Shen, Tetsu Sato, Shohei Koyama, Kaiji Uchida, Tappei Tanabe, Shinya Takaishi, Masahiro Yamashita, Hiroaki Iguchi

**Affiliations:** Department of Chemistry, Graduate School of Science, Tohoku University 6-3 Aza-Aoba, Aramaki Sendai 980-8578 Japan h-iguchi@tohoku.ac.jp; School of Materials Science and Engineering, Nankai University Tianjin 300350 P. R. China

## Abstract

Electrical conduction among metallocycles has been unexplored because of the difficulty in creating electronic transport pathways. In this work, we present an electrocrystallization strategy for synthesizing an intrinsically electron-conductive metallocycle, [Ni_6_(NDI-Hpz)_6_(dma)_12_(NO_3_)_6_]·5DMA·*n*H_2_O (PMC-hexagon) (NDI-Hpz = *N*,*N*′-di(1*H*-pyrazol-4-yl)-1,4,5,8-naphthalenetetracarboxdiimide). The hexagonal metallocycle units are assembled into a densely packed ABCABC… sequence (like the fcc geometry) to construct one-dimensional (1D) helical π-stacked columns and 1D pore channels, which were maintained under the liberation of H_2_O molecules. The NDI cores were partially reduced to form radicals as charge carriers, resulting in a room-temperature conductivity of (1.2–2.1) × 10^−4^ S cm^−1^ (pressed pellet), which is superior to that of most NDI-based conductors including metal–organic frameworks and organic crystals. These findings open up the use of metallocycles as building blocks for fabricating conductive porous molecular materials.

## Abstract 2

Intrinsically electron-conductive metallocycle was synthesized. π-Radicals play a key role in constructing π-stacked columns among molecular hexagons and achieving high electrical conductivity over 10^−4^ S cm^−1^ in polycrystalline pellet.

## Graphical abstract

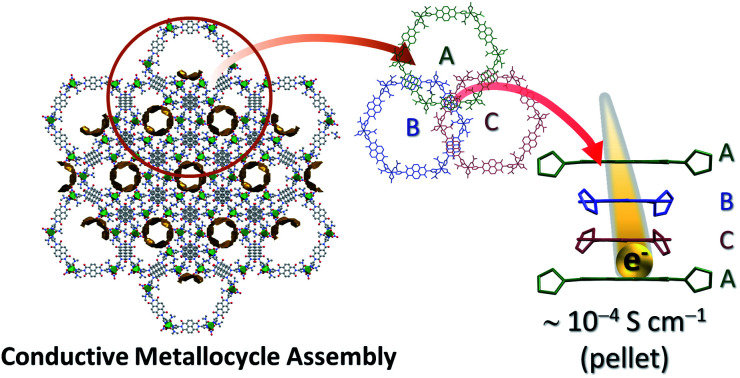

## Introduction

Giant macrocycles have attracted a great deal of attention due to their distinctive molecular structures and diverse functions.^1^ Coordination-driven self-assembly is a simple but powerful approach towards synthesizing giant metal–organic macrocycles (so-called metallocycles).^2^ Given the high designability of organic linkers and their various combinations with metal ions, metallocycles have provided a wide variety of molecular architectures such as rings,^3,4^ polygons,^5–8^ polyhedra,^7–9^ and nanotubes,^10–14^ in tandem with their multifunctional nature. For instance, anticancer ability,^15^ drug delivery,^16^ chirality recognition,^17^ and other molecular responsive functions^3,11,18^ have been realized by the flexible cavity of metallocycles. Furthermore, organic linkers with unique electronic properties have been key to fabricating functional metallocycles such as luminescent liquid crystals,^19^ asymmetric catalysts,^20^ and artificial light-harvesting systems.^21^ Although some metallocycles are not isolable in solution, solid-state functions have also been a subject of active research recently. To date, single-molecule magnet behavior,^10,22^ solid-state luminescence,^23^ heterogeneous catalytic behavior,^24^ gas storage,^25^*etc.*, have been reported in crystalline metallocycle assemblies. In this context, engendering electron transfer between assembled metallocycles is promising for providing conductivity as a new function of metallocycles, and moreover, it can be an alternative approach to design conductive porous crystals such as metal–organic frameworks (MOFs).^26^ However, only the hole mobility of dipyrrin-based polygons has been reported as the charge-transport property in metallocycles.^27^ Intrinsic conductivity, which requires both rich charge carriers and through-space conduction pathways among metallocycles, has not been realized thus far.

Electrocrystallization is a reasonable method to fulfill the above requirements, because the unpaired electrons (π-radicals) generated on π-conjugated planes by a redox reaction can be charge carriers and also induce an attractive interaction between π-conjugated planes. It has been widely used for the syntheses of organic conductors^28^ and, more recently, porous molecular conductors (PMCs),^29,30^ which are porous crystals consisting of linear coordination polymers with a conductive π-stacked array of *N*,*N*′-di(4-pyridyl)-1,4,5,8-naphthalenetetra-carboxdiimide (NDI-py) linkers. The NDI core is a well-known electron acceptor used in various research fields^31^ such as self-assembly,^32^ molecule sensors^33^ and molecular electronics,^34^ whereas its application in intrinsic electron conductors is still in the early stage of development.

Herein, we report an electrically conductive metallocycle synthesized by electrocrystallization for the first time. The coordination-driven self-assembly of hexagonal metallocycle units and the columnar self-assembly of π-conjugated organic linkers occurred together in the electrocrystallization process. In this work, pyrazole was selected as the terminal group of the organic linker because of their preferential construction of various bent coordination polymers^35^ and metallocycles.^36^

## Results and discussion

### Synthesis and discrete metallocycle structure

*N*,*N*′-Di(1*H*-pyrazol-4-yl)-1,4,5,8-naphthalenetetracarboxdi-imide (NDI-Hpz) and Ni(NO_3_)_2_·6H_2_O were dissolved in *N*,*N*-dimethylacetamide (DMA) with a small portion of H_2_O. Electrocrystallization was carried out by applying a constant direct current of 20 μA to the mixed solution at room temperature (RT). Needle-like tiny black crystals of [Ni_6_(NDI-Hpz)_6_(dma)_12_(NO_3_)_6_]·5DMA·*n*H_2_O (hereafter abbreviated as PMC-hexagon) were obtained from the cathode in 12 hours.

**Fig. 1:**
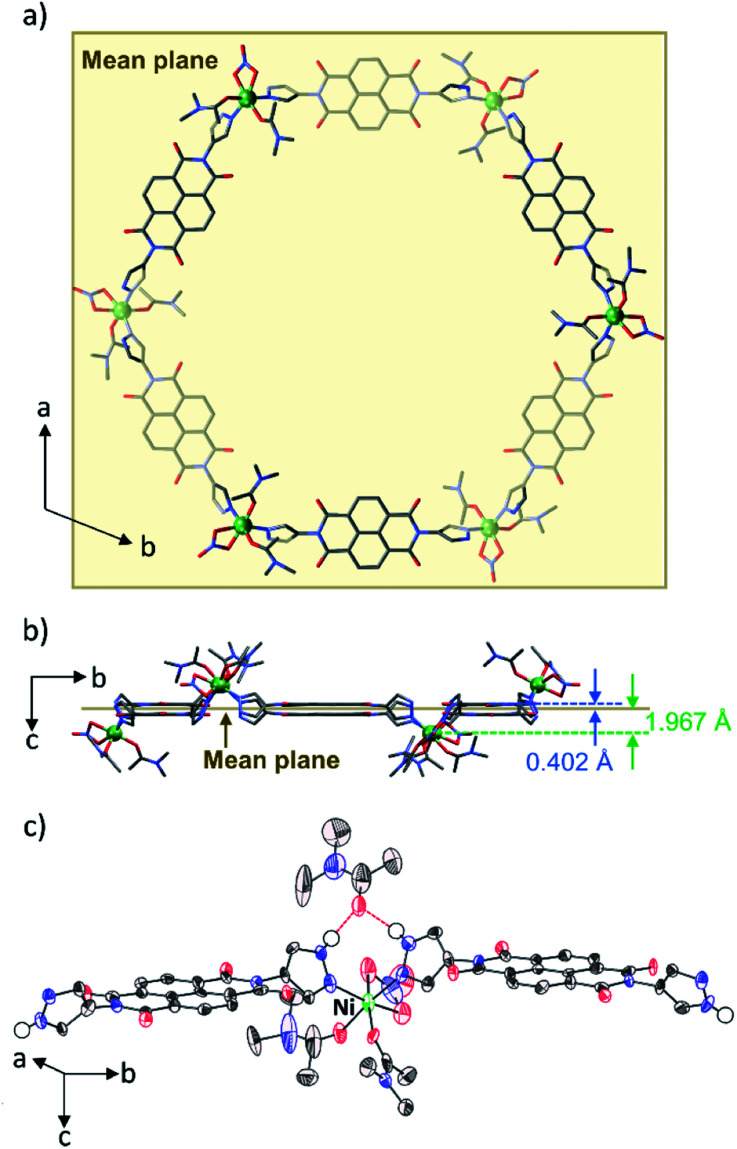
(a) Crystal structure of the hexagonal metallocycle in PMC-hexagon. The atoms under the mean plane of the hexagon are shaded. (b) Perspective view of the hexagonal metallocycle along the *a* axis showing the deviation of Ni^2+^ ions and the centroids of NDI cores from the mean plane of the hexagon. (c) Thermal ellipsoid plot of the ligands around Ni^2+^ ions including hydrogen-bonded DMA. Hydrogen bonds between the H atom of the pyrazole groups and the DMA are represented as red dashed lines. Other H atoms are omitted for clarity. Ni green, O red, N blue, and C black.

Single-crystal X-ray diffraction (SXRD) analysis revealed that PMC-hexagon crystallized in the trigonal space group *R*3̄ (Table S1†) with merohedral twinning. Remarkably, the hexagonal metallocycle structure [Ni_6_(NDI-Hpz)_6_(dma)_12_(NO_3_)_6_] constructed from head-to-tail connection between Ni^2+^ ions and NDI-Hpz linkers was elucidated as shown in Fig. 1a and b. A Ni^2+^ ion is coordinated by two pyrazole groups, two DMA molecules in cis geometry, and a bidentate NO_3_^−^ ion. As a result, the Ni^2+^ ion forms nearly octahedral coordination geometry (Fig. 1c). The positions of Ni^2+^ centres and the centroids of NDI cores deviate from the mean plane of the hexagon (1.967 Å and 0.402 Å, respectively), resulting in a pucker of the 6-membered metallocycle (Fig. 1b). The slightly large coordination-bond angle (∠N–Ni–N = 95.9(3)°) and the flexible dihedral angles between the NDI core and each pyrazole plane (60.39° and 62.79°) enable the puckered hexagon structure. In addition, a non-coordinating DMA molecule is located between two pyrazole groups with N–H⋯O(=C)⋯H–N hydrogen bonds (Fig. 1c). Since its occupancy is 84%, the total number of hydrogen-bonded DMA molecules is calculated to be five per metallocycle, in accord with the integration ratio in the ^1^H NMR spectrum (Fig. S7, ESI†).

### Packing structure of the metallocycle

**Fig. 2:**
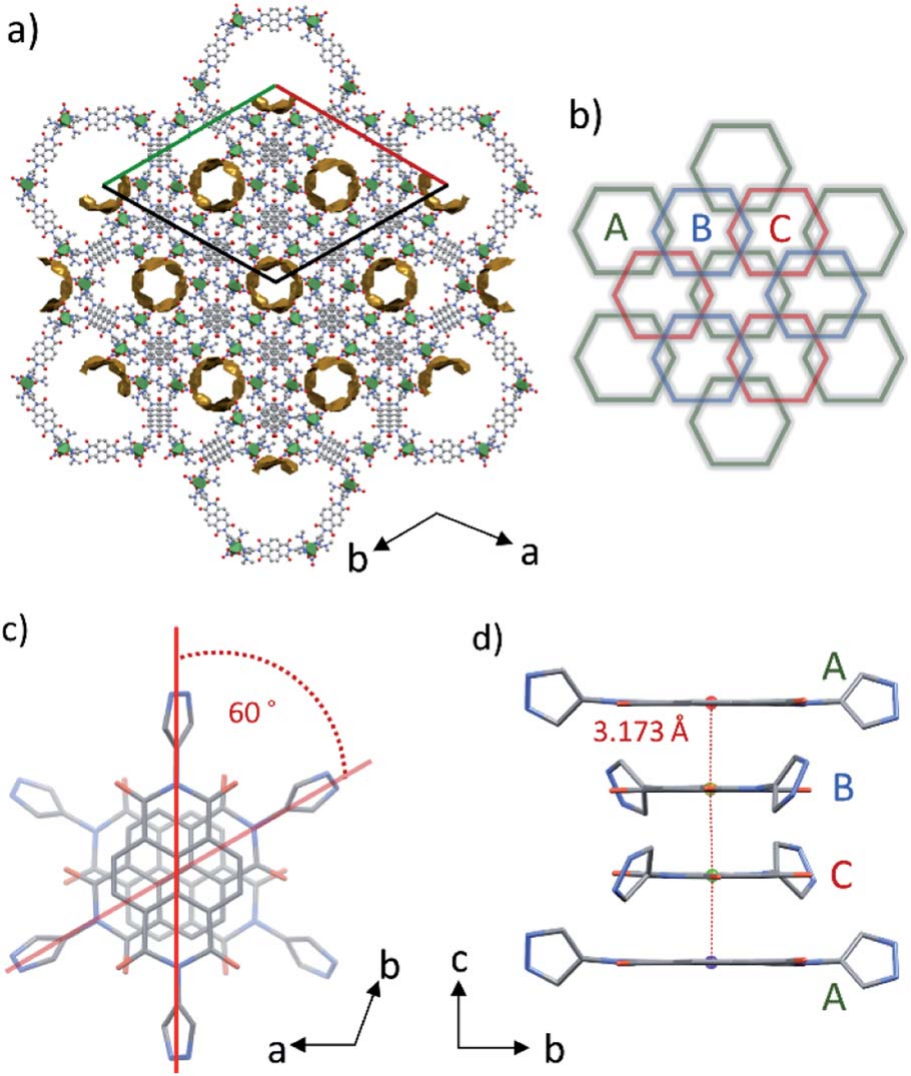
(a) Crystal packing structure along the *c* axis for PMC-hexagon. Coordination sphere around the Ni ion is represented as a green octahedron. The 1D voids of approximately 14.2% of the unit cell volume are mapped by the contact surface (yellow) of a probe with a radius of 1.4 Å. (b) Schematic illustration of the packing mode of hexagons densely stacked with the ABC… sequence. (c) Helical π-stacked column of NDI-Hpz projected along the *c* axis. (d) Side view of the 1D π-stacked column with the interplanar distance.

These discrete hexagonal metallocycles stack atop one another at the NDI cores. The crystal packing structure of PMC-hexagon (Fig. 2a) displays the hexagonal arrangement of the one-dimensional (1D) helical π-stacked columns and 1D pore channels extended along the crystallographic *c* axis. It is constructed from the dense packing of hexagonal metallocycles with an alternating sequence of ABCABC… (Fig. 2b). This stacking manner, which is similar to the face-centered cubic (fcc) geometry, has rarely been observed in the self-assembly of metallocycles.^37^ The helical stacking mode of NDI cores in 60° increments (Fig. 2c) plays a crucial role in overlapping the sides of hexagons and forming the ABCABC… sequence. Although the pyrazole group can form a variety of metallocycle-based polygons,^36^ hexagons were selectively crystallized probably because of their compatibility with the helical stacking mode mentioned above.

The interplanar distance between adjacent NDI-Hpz ligands along the stacking axis is 3.173 Å (1/3 of the unit cell length c), which is shorter than those (>3.3 Å) between typical neutral NDI cores,^32*a*,38^ indicating the attractive interaction derived from NDI radicals (NDI˙^−^) (Fig. 2d).^29,30^ The transfer integral between the adjacent LUMO of NDI-Hpz linkers along the stacking axis was calculated to be 315 meV. It is significantly larger than those of neutral NDI-based organic semiconductors (typically less than 100 meV),^39^ reflecting the strong π-stacking interaction in PMC-hexagon.

**Fig. 3:**
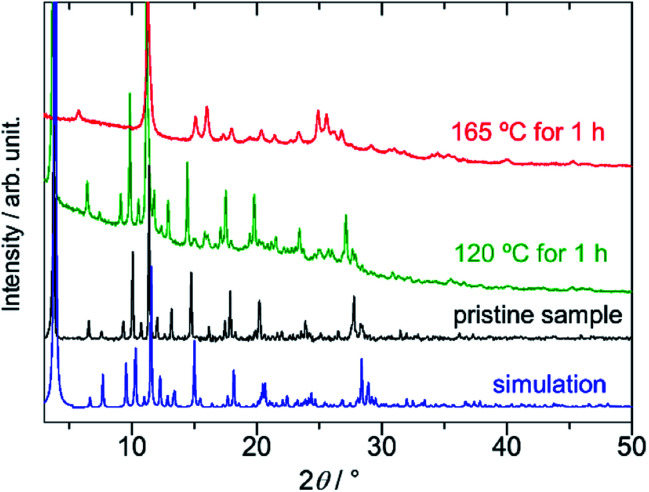
Powder X-ray diffraction (PXRD) patterns of PMC-hexagon (simulated from the crystal structure: blue, pristine sample: black, heated at 120 °C for an hour: green, and heated at 165 °C for an hour: red).

The dense packing decreases their cavities, resulting in a small void space (14.2% of the total volume). On the basis of elemental analysis, the 1D pore channel is likely to accommodate H_2_O molecules (see the Experimental section), whereas their exact number was uncertain because the liberation of H_2_O molecules occurred even at RT (Fig. S1, ESI†). After heating PMC-hexagon at 120 °C for an hour, the powder X-ray diffraction (PXRD) pattern (Fig. 3) and the number of contained DMA molecules estimated from the integration ratio in the ^1^H NMR spectrum (Fig. S8, ESI†) were almost unchanged, though a weight loss was observed in thermogravimetry analysis (TGA). These results suggest that the crystal structure was maintained under the liberation of H_2_O molecules in the 1D pore channel. The heating of PMC-hexagon at 165 °C for an hour induced the liberation of DMA molecules and the deterioration, as suggested by the change of the ^1^H NMR spectrum (Fig. S9, ESI†) and the PXRD pattern (Fig. 3). Thus, it is difficult to obtain a desolvated state without deterioration in contrast to previously reported PMC with a similar π-stacked analog [Cd(NDI-py)(OH_2_)_4_](NO_3_)_1.3±0.1_·*n*DMA (PMC-1).^29^ However, it is notable that PMC-hexagon retained the crystal structure under the liberation of H_2_O molecules despite the lack of a coordination polymer network like MOFs. This robustness should originate from the dense packing supported by the strong π-stacking interaction.

Although the void volume of an individual 1D pore channel is comparable between PMC-hexagon and PMC-1 (approx. 820 Å^3^ par channel in the unit cell), PMC-hexagon was more air-sensitive than PMC-1, probably because the fast liberation of H_2_O molecules from the channel promotes the access of oxygen molecules to NDI˙^−^ species. The nitrogen gas adsorption in the micropore of PMC-hexagon has not been observed (Fig. S10, ESI†), presumably because of the high-vacuum conditions in the activation process. The partial deterioration due to the liberation of DMA molecules probably occurred at least on the surface of the solid during the vacuuming and disturbed the insertion of nitrogen molecules into channels, as suggested by the PXRD pattern (Fig. S11, ESI†) and the ^1^H NMR spectrum (Fig. S12, ESI†).

### Physical properties of PMC-hexagon

The existence of NDI˙^−^ was confirmed by a sharp signal (*g* = 2.00354) in the electron spin resonance (ESR) spectrum (Fig. S2, ESI†). The contribution of NDI˙^−^ species to the magnetic properties of PMC-hexagon is very small, because the Ni^2+^ ion has a larger spin magnetic moment (*S* = 1) and the antiferromagnetic interaction and/or partial dimerization along the π-stacked column decreases the total magnetic moment of NDI˙^−^ species. We estimated the number of effective NDI˙^−^ species in the range from 10 to 15% by fitting the temperature dependence of the *χ*_M_*T* value (*χ*_M_: molar spin susceptibility and *T*: temperature) with the aid of quantum calculations (see Fig. S3† and explanation in the ESI†).

**Fig. 4:**
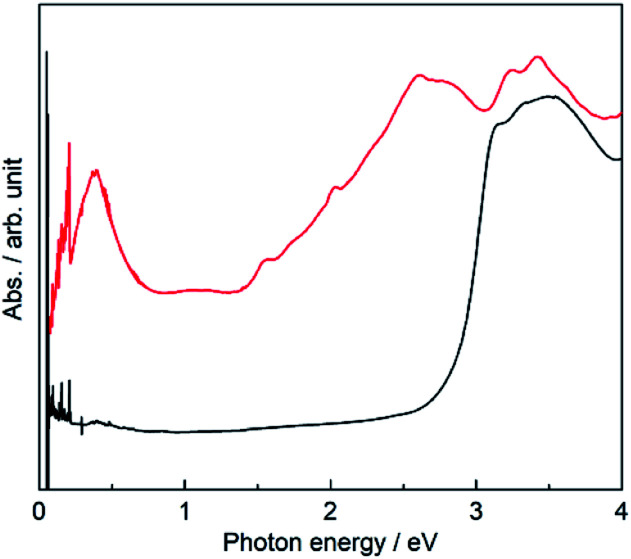
Solid-state absorption spectra of PMC-hexagon (red) and neutral NDI-Hpz (black) dispersed in KBr pellets.

To investigate the electronic state in detail, the solid-state absorption spectra of PMC-hexagon and NDI-Hpz dispersed in KBr pellets were acquired. The whole process from the synthesis to the measurement was carried out under an argon atmosphere in order to avoid the air oxidation of radicals. Both spectra display strong absorption bands around 3.3 eV, which are assignable to intramolecular π–π* transition in neutral NDI (NDI^0^) cores (Fig. 4).^29,30,40^ Additional absorption bands were observed in the spectrum of PMC-hexagon as well as reported NDI˙^−^ species. The strong band in the range of 2.5–2.8 eV and weak bands around 2.0 eV and 1.5 eV are ascribed to the intramolecular electron transition of NDI˙^−^.^29,41,42^ The weak broad band around 1 eV and the relatively strong band around 0.4 eV are ascribed to the charge transfer (CT) between two NDI˙^−^ cores and that from NDI˙^−^ to NDI^0^ cores, respectively.^29,30,40,43^ The existence of NDI^0^ species in PMC-hexagon is inconsistent with the formula, which indicates that the charge of NDI-Hpz is −1. Whilst the concentration of NDI^0^ in the crystal could not be determined with accessible analytical methods, we believe that partial deprotonation of the Hpz group satisfies charge neutrality in this system. The partial absence of the hydrogen-bonded DMA molecules (84% occupancy) may reflect the loss of hydrogen donors due to the partial deprotonation.

**Fig. 5:**
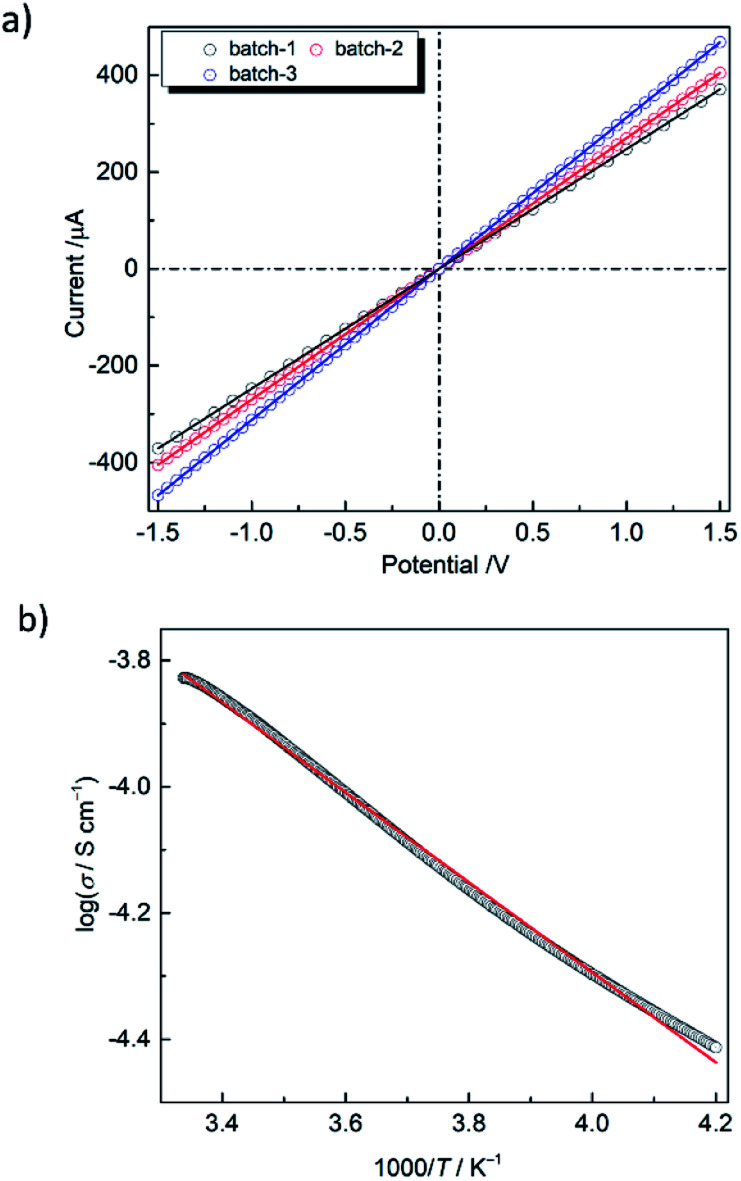
(a) Current–voltage (*I*–*V*) characteristics of PMC-hexagon in three different pellets at RT. (b) Temperature dependence of the electrical conductivity (*σ*) of PMC-hexagon fitted with the Arrhenius equation (red line).

The low energy CT band suggests that PMC-hexagon is a narrow-gap semiconductor. Since the single crystals of PMC-hexagon were too small to measure the electrical conductivity (*σ*), the measurements were carried out on 3 mm*ϕ* pressed pellets. The current–voltage (*I*–*V*) plots indicate the ohmic characteristic (*V* = *RI*, where *R* is the electrical resistance) as shown in Fig. 5a. The *σ* of PMC-hexagon at RT was calculated to be (1.2–2.1) × 10^−4^ S cm^−1^. It is 10 times higher than the *σ* of PMC-1, which was newly prepared and measured under the same inert conditions in this work (Fig. S4, ESI†). Note that the *σ* of PMC-1 in previous work ((1.5–7.6) × 10^−6^ S cm^−1^)^29^ was obtained under incompletely inert conditions and is lower than that obtained in the present work ((1.4–2.0) × 10^−5^ S cm^−1^ in pressed pellets), indicating that the avoidance of air oxidation is important to properly measure *σ* in NDI-based conductors. The π-stacked columns in PMC-hexagon are surrounded by Ni complexes and are away from 1D pore channels, freeing it from the perturbation of conduction carriers by disordered molecules in the pores. It may be one of the reasons for the 10-fold increase in conductivity compared to PMC-1. The temperature dependence of *σ* (Fig. 5b) shows the decrease in *σ* with decreasing temperature, namely semiconducting behavior. The activation energy (*E*_a_) is determined to be 0.148 eV by fitting the data with the Arrhenius equation *σ* = *σ*_0_ exp(−*E*_a_/*kT*), where *σ*_0_ is the prefactor, *k* is the Boltzmann constant and *T* is the temperature. The *E*_a_ of PMC-hexagon is smaller than that in PMC-1 (0.25 eV),^29^ supporting the highly conducting nature of PMC-hexagon. To date, the reported *σ* of NDI-based conductive MOFs/coordination polymers^29,44^ and organic crystals^45^ have been in the range from 10^−7^ to 2.0 × 10^−5^ S cm^−1^ for pressed pellets and up to 3.3 × 10^−3^ S cm^−1^ for single crystals. The *σ* of a pressed pellet of 1D conductors is typically smaller than that of the single crystal by two to three orders of magnitude. Therefore, PMC-hexagon is one of the most conductive NDI-based crystals. Although the *σ* of PMC-hexagon is lower than the record values for conductive MOFs with through-space conduction pathways (10^−4^ to 0.05 S cm^−1^ for lanthanide-hexahydroxytriphenylene-based MOFs),^46^ it is higher than that of other π-stacked MOFs (*e.g.* 10^−9^ to 7.6 × 10^−5^ S cm^−1^ (pressed pellet) for MOFs with a TTF moiety^47^). Moreover, the high *σ* (above 0.1 S cm^−1^) observed in some NDI-based polymers^48^ implies that we have much room to improve the conductivity of NDI-based metallocycles, which can be an alternative building block to construct conductive porous molecular crystals.

## Conclusions

Intrinsically electron-conductive metallocycle PMC-hexagon was synthesized by using electrocrystallization. The hexagonal metallocycles were densely π-stacked in a face-centered cubic (fcc)-like geometry (ABCABC… sequence) to afford 1D helical π-stacked conductive columns and 1D pore channels. The radical at the NDI core (NDI˙^−^) provides quite a short interplanar distance (3.173 Å) and conducting carriers, resulting in a high electrical conductivity ((1.2–2.1) × 10^−4^ S cm^−1^ at RT) even in the polycrystalline pellet sample. Despite the lack of a coordination polymer network, the dense packing of hexagonal metallocycles provides a robust structure, which was maintained under the liberation of H_2_O molecules in the pore channels. The elimination of DMA molecules induced the deterioration and disturbed the nitrogen adsorption to pore channels. Although the improvement of the robustness and the conductivity remains to be addressed, the concept of using a metallocycle as a building block provides a new platform for developing electrically conductive porous molecular materials.

## Experimental

### Materials and characterization

All purchased chemicals were used without further purification. ^1^H NMR spectra were recorded on a Bruker AVANCE 500 spectrometer at RT. ^1^H NMR chemical shifts were referenced internally to residual solvent resonances. Deuterated solvents were obtained from Wako and used as received. Electrocrystallization was performed using a direct current (DC) multisource, YAZAWA CS-12Z, and 0.3 mm electrodes of platinum-iridium alloy wires (Pt : Ir = 80 : 20). All elemental analyses were performed at a J-SCIENCE LAB, JM-11 at the Research and Analytical Centre, Tohoku University. Japan.

### Synthetic procedures

#### Synthesis of 4-nitropyrazole

Pyrazole (2.05 g, 29.6 mmol) was dissolved in H_2_SO_4_ (98%) (6 mL) and cooled to 0 °C. HNO_3_ (*ca.* 67%) (1.2 mL) was then added dropwise over 5 minutes. The mixture was then heated to 60 °C and stirred for 2 hours. The mixture was then cooled to 0 °C, the acidic solution was poured into *ca.* 100 g of ice and then stirred for 10 minutes upon which, a white precipitate was formed. The mixture was neutralized with Na_2_CO_3(aq)_, extracted with ethyl acetate (100 mL × 3), washed with brine solution, and concentrated to yield a white crystalline powder. The product was recrystallized from ethyl acetate and dried *in vacuo*. Yield: 2.059 g (61.2%). ^1^H NMR (DMSO-*d*_6_, 500 MHz): *δ* 13.94 (s, 1H), 8.87 (s, 1H), 8.25 (s, 1H) ppm. Elemental analysis, found: C, 31.95; H, 2.7; N, 37.2. Calc. for C_3_H_3_N_3_O_2_: C, 31.85; H, 2.7; N, 37.2.

#### Synthesis of 4-aminopyrazole

4-Nitropyrazole (2.867 g, 25.4 mmol) was dissolved in anhydrous EtOH (100 mL) in a Schlenk flask and degassed under N_2_ for 1 hour. Pd/C powder (10% w/w) was then added and the mixture was further degassed for 1 hour. The flask was then put under reduced pressure and a H_2_ gas-filled balloon (1 atm) was then connected. The reaction mixture was then stirred vigorously for 24 hours. The H_2_ gas was refilled upon depletion during the reaction. Upon completion of the reaction, the Pd/C powder was separated *via* filtration, and the volume of the filtrate was reduced to yield a reddish oil which partially solidified upon cooling to 5 °C overnight. The product is hygroscopic in ambient air. Yield: 2.04 g (95.5%). ^1^H NMR (D_2_O, 500 MHz): *δ* 7.28 (s, 2H) ppm. Elemental analysis, found: C, 42.1; H, 6.1; N, 48.7. Calc. for C_3_H_5_N_3_: C, 43.35; H, 6.1; N, 50.6. (Note: discrepancy is due to the hygroscopic nature of the product).

#### Synthesis of *N*,*N*′-di(1*H*-pyrazol-4-yl)-1,4,5,8-naphthalenetetra-carboxdiimide (NDI-Hpz)

4-Aminopyrazole (654 mg, 7.78 mmol) and 1,4,5,8-naphthalenetetracarboxylic dianhydride (1.011 g, 3.97 mmol) were suspended in DMF (40 mL) and degassed for 20 minutes. The reaction was put under N_2_ and refluxed for 24 hours. Upon cooling to RT, 80 mL of H_2_O was added to the mixture while stirring to yield a yellow/maroon precipitate. The precipitate was filtered, washed with acetone (30 mL × 3), and dried under air. The product was recrystallized from *N*,*N*-dimethylacetamide (DMA) and H_2_O to yield a light-yellow powder. Single crystals of NDI-Hpz were obtained by slow diffusion of MeOH to a saturated NDI-Hpz solution in DMA. Yield: 1.35 g (43.6%). ^1^H NMR (DMSO-*d*_6_, 500 MHz; see Fig. S5, ESI†): *δ* 13.10 (s, 2H), 8.71 (s, 4H), 7.95 (s, 2H), 7.63 (s, 2H) ppm. Elemental analysis, found: C, 60.15; H, 2.7; N, 21.0. Calc. for C_20_H_10_N_6_O_4_: C, 60.3; H, 2.5; N, 21.1.

#### Synthesis of [Ni_6_(NDI-Hpz)_6_(dma)_12_(NO_3_)_6_]·5DMA·*n*H_2_O (PMC-hexagon)

For the synthesis of bulk PMC-hexagon, NDI-Hpz (20 mg, 0.050 mmol) was suspended in DMA (6 mL) and heated until all solids were dissolved. Upon cooling to RT, the solution was degassed under N_2_ for about 30 minutes. To the solution, Ni(NO_3_)_2_·6H_2_O (29.1 mg, 0.100 mmol) and H_2_O (20 μL) were added and sonicated for around 3 minutes. The solution was then put under a constant current of 20 μA. A black microcrystalline product began to appear around the cathode overnight. After the reaction was continued for about 3 days, the product was carefully removed from the cathode and washed with degassed DMA solution under an N_2_ atmosphere and dried in glovebox filled with an Ar atmosphere. To obtain single crystals suitable for structural analysis, a degassed DMA solution (3 mL) containing NDI-Hpz (2.6 mg, 6.5 μmol), Ni(NO_3_)_2_·6H_2_O (3.8 mg, 13 μmol) and H_2_O (20 μL) was prepared. After a constant current of 20 μA was applied to the solution overnight, black needle-like crystals of PMC-hexagon were obtained. Elemental analysis, found: C, 47.2; H, 4.9; N, 16.8. Calcd for C_188_H_237_N_59_Ni_6_O_71_: C, 46.9; H, 5.0; N, 17.2 (assumed that *n* = 12). (Note: due to the liberation of H_2_O molecules at RT, the number of lattice H_2_O molecules depends on the elapsed time since the sample filtration).

## Data availability

Detailed data including general information, characterization, crystallographic parameters, magnetism, electrical conductivity and computational methods are available in the ESI.†

## Author contributions

H. I. conceived and designed the project. M. C. carried out the synthesis, characterization, crystal structure analysis and measuring of the physical properties of PMC-hexagon. R. M. contributed to the design of the experiment, syntheses of ligands and PMC-hexagon, and preliminary crystal structure analysis. Y. S. contributed to the measurement and analysis of electrical conductivity. T. S. and K. U contributed to the magnetic measurements and the analyses using calculation and fitting. K. U. also contributed to the nitrogen adsorption measurement. S. K. performed the theoretical calculation of the transfer integral. T. T. contributed to measurements and characterization related to nitrogen sorption isotherms and the thermogravimetry analysis. S. T., M. Y. and H. I. supervised this work and carried out discussions. M. C. and H. I. wrote the paper.

## Conflicts of interest

There are no conflicts to declare.

## Supplementary Material

SC-013-D2SC00447J-s001

SC-013-D2SC00447J-s002
