# Supplementary material for: An electrically conductive metallocycle: densely packed molecular hexagons with π-stacked radicals
Source: Chem Sci. 2022 Apr 1;13(17):4902–8. doi: 10.1039/d2sc00447j (PMC9067574; doi:10.1039/d2sc00447j)
Supplement: SC-013-D2SC00447J-s001 [file SC-013-D2SC00447J-s001.pdf]

Electronic Supplementary Material (ESI) for Chemical Science.
This journal is © The Royal Society of Chemistry 2022

Supporting Information

# An electrically conductive metallocycle: densely packed molecular hexagons with π-stacked radicals

Mengxing Cui,[a] Ryuichi Murase,[a] Yongbing Shen,[a] Tetsu Sato,[a] Shohei Koyama,[a] Kaiji Uchida,[a] Tappei Tanabe,[a] Shinya Takaishi,[a] Masahiro Yamashita,[a,b] and Hiroaki Iguchi*[a]

[a]Department of Chemistry, Graduate School of Science, Tohoku University, 6-3 Aza-aoba, Aramaki, Aoba-ku, Sendai 980-8578, Japan.

[b]School of Materials Science and Engineering, Nankai University, Tianjin 300350, P. R. China.

**Table of Contents**

General information……………………………………………………………………………………………..2
Crystallographic information of **PMC-hexagon** (Table S1)……………………………………………………………4
Thermogravimetry analysis (TGA) of **PMC-hexagon** (Fig. S1)………………………………………………………4
Solid-state electron spin resonance (ESR) spectrum of **PMC-hexagon** (Fig. S2)………………………………..5
Temperature dependence of $\chi_M T$ value of **PMC-hexagon** and discussion on magnetism (Fig. S3, Tables S2, S3) ……………………………………………………………………………………………………………5
Current-voltage (*I*-*V*) characteristics of PMC-1 (Fig. S4) ……………………………………………………………7
$^1$H NMR spectra of NDI-Hpz and **PMC-hexagon** in DMSO-$d_6$ (Fig. S5–S9)………………………………………8
$N_2$ sorption isotherm of **PMC-hexagon** (Fig. S10)……………………………………………………………………11
PXRD patterns of **PMC-hexagon** after the measurement of $N_2$ sorption isotherm (Fig. S11)………………11
$^1$H NMR spectrum of **PMC-hexagon** after the measurement of $N_2$ sorption isotherm (Fig. S12)………………12
References………………………………………………………………………………………………………..13

## General Information

IR spectra were collected with KBr pellets on a JASCO FT/IR-4200 spectrometer at room temperature (RT). UV-Vis-NIR absorption spectra were collected with KBr pellets on a JASCO V-670 spectrophotometer at RT. KBr pellets were prepared in a glovebox (MBRAUN UNILAB1200/780) filled with Ar gas, and then sealed in a custom cell for IR and UV-Vis-NIR measurement under an inert atmosphere. Thermogravimetry (TGA) analysis was carried out on a SHIMADZU DTG-60H with a heating rate of 5 °C/min under a constant nitrogen gas flow (0.1 L/min). The ESR spectrum was acquired by using a JEOL JES-FA100. The static magnetic susceptibility was measured on polycrystalline sample in the temperature range of 1.8–300 K using a superconducting quantum interference device (SQUID) magnetometer. The calculation of the intrinsic diamagnetic correction was conducted using common Pascal's constants.[1]

**Single-crystal X-ray diffraction (SXRD).** The diffraction data for **PMC-hexagon** were collected on a Rigaku XtaLAB AFC10 diffractometer with a HyPix-6000HE hybrid pixel array detector, graphite monochromated Mo Kα radiation ($\lambda$ = 0.7107 Å) and a cryogenic equipment GN-2D/S. The crystal structure was solved using direct methods (Sir2019[2]), followed by Fourier synthesis. Structure refinement was carried out using full-matrix least-squares procedures with SHELXL[3] on $F^2$ in the Yadokari-XG 2009 software.[4] The SQUEEZE method was applied to remove the electron density in 1D pore channels, because it was too dispersed to fabricate a meaningful molecular structure.[5] The CCDC number of crystallographic data for **PMC-hexagon** was assigned to CCDC 2091485.

**Electrical conductivity measurement.** Polycrystalline powder of **PMC-hexagon** was loaded into an alumina ring that was set on the one of the pellet dices. Then the other pellet dice was inserted to the alumina ring and the pressure was applied by using Specac Mini-Pellet Press. The 3 mmΦ pressed pellet prepared in the insulating alumina ring was connected to Agilent E5260A via the pellet dices, which act as electrode, and the current-voltage (*I*-*V*) characteristic was collected. Synthesis of **PMC-hexagon**, pellet fabrication and the *I*-*V* measurement of the pellets were carried out in a glovebox (MBRAUN UNILAB1200/780) filled with Ar gas.

The temperature dependence of the electrical conductivity was measured in a liquid He cryostat of a Quantum Design PPMS (Physical Property Measuring System) model 6000 by using the two-probe method in direct current (DC) mode with Keithley sourcemeter model 2611. The cooling rate was 1 K/min. The 3 mmΦ pressed pellet was prepared in the glovebox and connected to the sample puck by gold wires (20 µmΦ) and carbon paste (Dotite XC-12 in diethyl succinate) under air. Then, the carbon paste was dried in the glovebox.

**Computational method.** Amsterdam Modeling Suite (AMS) packages[6,7] were applied for the calculations of transfer integral of **PMC-hexagon**. The transfer integral between adjacent NDI-Hpz molecules was investigated by the B3LYP-D3/TZP method[8] without structural optimization. To estimate the g-factor of $Ni^{2+}$ ion, quantum calculations were conducted by using the ORCA4.2.1 package program suite.[9,10] CASSCF(8,5) calculations using the minimal active spaces comprising only d-electrons were performed. Cartesian coordinates of the initial geometry was used from crystal structure with replacing NDI-core to hydrogen atom. The def2-SVP basis set and RIJCOSX was used for all atoms. To consider the effect of the dynamic correlation, NEVPT2 was employed on top of the converged CASSCF wave function.[11]

**Adsorption measurement.** $N_2$ sorption isotherm was performed using microtracbel Belsorp Max with G1 grade (>99.9999 %) gas. Solid **PMC-hexagon** was activated by heating at 120 °C for an hour under high-vacuum condition with turbo molecular pump. Sorption measurement was performed by volumetric method, and the volume of adsorbed gas was calculated from the ideal gas equation with second virial coefficient ($H_2$: 3.436 × $10^{-9}$, $N_2$: –4.264 × $10^{-7}$). $N_2$ sorption isotherms were measured for **PMC-hexagon** at 77 K. Temperature control during sorption measurement was done by using Dewar bottle. Adsorption isotherm was measured in the pressure range of $P/P_0$ = $10^{-5}$–0.997 ($P_0$ = 101.325 kPa) and desorption isotherm was measured down to $P/P_0$ = 0.1. The volume of sample space was measured after sorption measurement by using G2 grade (>99.999 %) He gas, and dead volume correction was applied to the raw data.

## Crystallographic information of PMC-hexagon

**Table S1.** Crystallographic information of **PMC-hexagon** with SQUEEZE treatment.

| Compound | **PMC-hexagon** |
|---|---|
| Chemical formula | $C_{188.19}H_{213.43}N_{59.05}NiO_{59.05}$ |
| *F*w (g/mol) | 4599.64 |
| *T* (K) | 120 |
| Crystal system | Trigonal |
| Space group | *R*-3 |
| *a* (Å) | 45.980(2) |
| *c* (Å) | 9.5196(7) |
| *V* (Å$^3$) | 17430(2) |
| *Z* | 3 |
| *ρ* calcd. (g/cm$^3$) | 1.315 |
| *μ* (mm$^{-1}$) | 0.564 |
| *F*(000) | 7189 |
| Crystal size (mm$^3$) | 0.17 x 0.02 x 0.02 |
| Radiation, wavelength | Mo Kα, 0.71073 Å |
| GOF on $F^2$ | 1.028 |
| $R_1$, $wR_2$ [$I > 2\sigma(I)$] | 0.0992, 0.2428 |
| $R_1$, $wR_2$ [all data] | 0.2263, 0.2908 |

## Thermogravimetry analysis (TGA) of PMC-hexagon

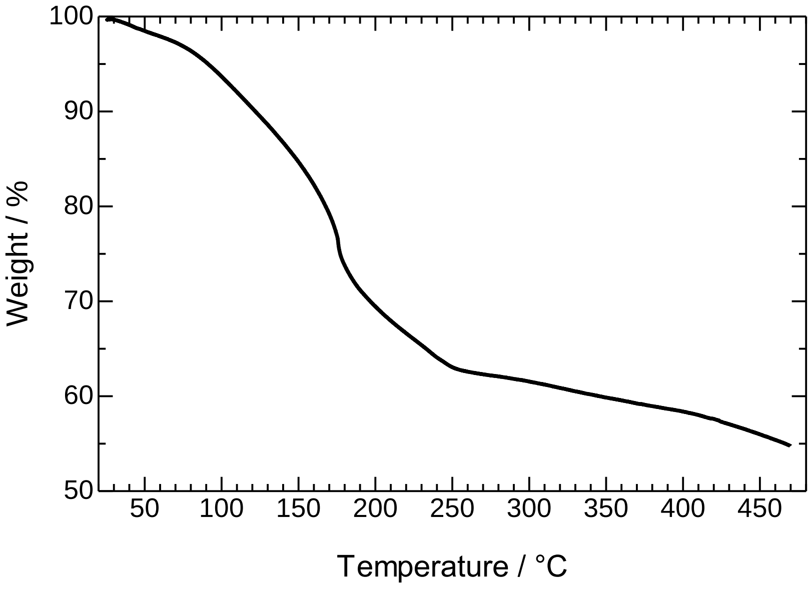


**Fig. S1.** Thermogravimetry analysis (TGA) of **PMC-hexagon** with a heating rate of 5 °C/min.

## Solid-state electron spin resonance (ESR) spectrum of PMC-hexagon

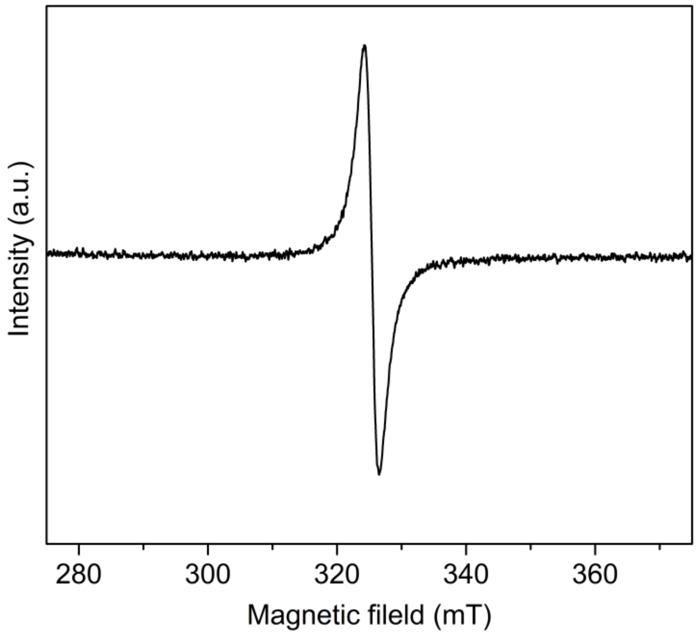


**Fig. S2.** Solid-state spin resonance (ESR) spectrum of **PMC-hexagon** measured at RT. A sharp signal ($g$ = 2.00354) implies the existence of radical at the NDI core. The signal from $Ni^{2+}$ spins was negligible at RT due to the broadening.

## Temperature dependence of $\chi_M T$ value of PMC-hexagon and discussion on magnetism

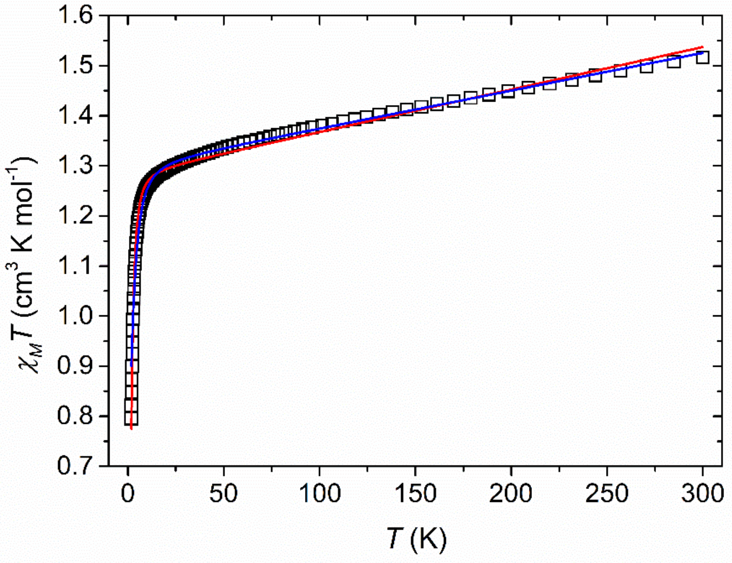


**Fig. S3.** Temperature dependence of $\chi_M T$ value ($\chi_M$: molar spin susceptibility, $T$: temperature) of **PMC-hexagon** at 1000 Oe. Red and blue curves represent the calculated data using [Eq.(1)] with isotropic g-factor ($g_{iso}$ = 2.23) and parameters listed in Table S3 (NEVPT2, red: $D > 0$, blue: $D < 0$).

The static magnetic property of **PMC-hexagon** was measured with a temperature range of 1.8–300 K as shown in Fig. S3. In the simple case, the $\chi_M T$ values of $S$ =1 spin ($Ni^{2+}$ ion) and $S$ =1/2 spin (NDI radical) are expected to 1.000 and 0.375 $cm^3$ K $mol^{-1}$, respectively at RT. Assuming that the amount of $NDI^{\bullet -}$ species is 84% of the total NDI cores from the occupancy of hydrogen-bonded DMA molecules, the total $\chi_M T$ value is expected to 1.315 $cm^3$ K $mol^{-1}$. In practice, however, the experimental $\chi_M T$ value (1.516 $cm^3$ K $mol^{-1}$ at 300 K) is higher than the expected value due to the contribution of several factors such as spin-orbit coupling, which is typically represented as the deviation of g-factor from 2. We first estimated the g-factors of $Ni^{2+}$ ion from the coordination geometry obtained by SXRD analysis. The quantum calculations (CASSCF and NEVPT2) using the ORCA4.2.1 package program was conducted and the results are shown in Table S2.

**Table S2.** Calculated factors based on the coordination geometry around $Ni^{2+}$ ion in **PMC-hexagon**.

| | CASSCF | NEVPT2 |
|---|---|---|
| $g_x$, $g_y$, $g_z$ | 2.293650, 2.314802, 2.330928 | 2.219871, 2.234914, 2.246589 |
| $g_{iso}$ | 2.313127 | 2.233792 |
| $D$[a]/cm$^{-1}$ | -4.622478 | -3.268454 |

[a] Zero-field splitting parameter.

Since the anisotropy of g-factors is very small, the isotropic g-factor ($g_{iso}$) was used for the next step. The temperature dependence of $\chi_M T$ value was fitted by [Eq.(1)] using least-square method with the fixed $g_{iso}$ and three variables (zero-field splitting parameter ($D$), temperature-independent paramagnetism (TIP) and molar ratio of effective $NDI^{\bullet -}$ species ($n$). The results of fitting parameters are listed in Table S3.

$$\chi_M T = 0.375n + TIP \cdot T + \frac{0.75 g_{iso}{}^2 \exp\left(-\frac{D}{kT}\right)}{3\left(1 + 2\exp\left(-\frac{D}{kT}\right)\right)} + \frac{2T \cdot 0.5212 g_{iso}{}^2 \left(1 - \exp\left(-\frac{D}{kT}\right)\right)}{3D\left(1 + 2\exp\left(-\frac{D}{kT}\right)\right)} \quad \text{[Eq. (1)]}$$

**Table S3.** Parameters optimized for fitting $\chi_M T$-$T$ plot using [Eq.(1)] with fixed $g_{iso}$ obtained by CASSCF and NEVPT2 quantum calculations.

| | CASSCF | NEVPT2 ($D$ > 0) | NEVPT2 ($D$ < 0) |
|---|---|---|---|
| $g_{iso}$(fixed) | 2.313 | 2.23 | 2.23 |
| $D$/cm$^{-1}$ | –6.49 | 4.02 | –6.97 |
| TIP/cm$^3$mol$^{-1}$ | 7.52×10$^{-4}$ | 8.47×10$^{-4}$ | 7.52×10$^{-4}$ |
| $n$ | –0.101 | 0.105 | 0.150 |

The fitting using $g_{iso}$ obtained by CASSCF calculation gave negative $n$ value. It is obviousely inconsistent with the existence of $NDI^{\bullet -}$ species confirmed by the ESR spectrum (Fig. S2). On the other hand, the fitting using $g_{iso}$ obtained by NEVPT2 calculation gave reasonable parameters. As shown in Fig. S3, the calculated curves using these parameters are in good agreement with the $\chi_M T$-$T$ plot. The $D$ values were so small that it is not suitable to determine their signs from the fitting. In both cases, the amount of effective $NDI^{\bullet -}$ species is in the range of 10 to 15%. These small $n$ values suggest the antiferromagnetic interaction between radical spins and/or spin quenching by partial dimerization of NDI cores.

## Current-voltage (*I*-*V*) characteristics of PMC-1

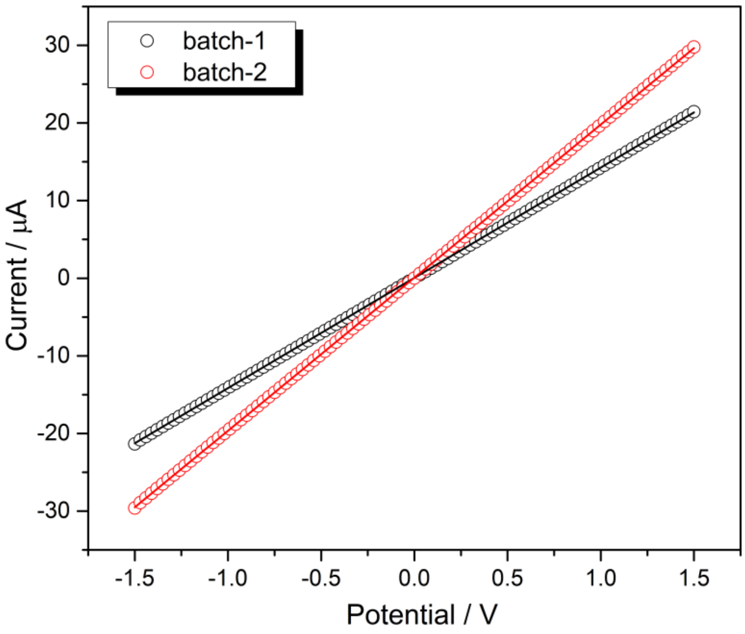


**Fig. S4.** Current-voltage (*I*-*V*) characteristics of PMC-1 measured in pressed pellets at RT. Whole process from the synthesis to the measurement was carried out under argon atmosphere as well as **PMC-hexagon** (Fig. 5a).

## $^{1}$H NMR spectra of NDI-Hpz and PMC-hexagon in DMSO-$d_6$

To estimate the amount of DMA molecules in the crystal, $^{1}$H NMR spectra of NDI-Hpz and **PMC-hexagon** (pristine and heated samples) dissolved in DMSO-$d_6$ solution were acquired at RT. A drop of conc. $H_2SO_4$ was added to dissolve **PMC-hexagon** perfectly. In this acidic condition, NDI-Hpz exists as protonated state, as supported by the single peak attributed to the hydrogen atoms in a protonated pyrazole group (**a** and **b** in Fig. S6). The amount of DMA molecules were calculated by comparing the integration values of **a** or **b** with **z** in Fig. S7 and S8, taking into consideration that **a** and **b** reflect four hydrogen atoms per a NDI-Hpz moleluce and **z** reflect three hydrogen atoms per a DMA molecule. The integration value of **z** was used rather than those of **x** and **y** because **z** is farther from the large residual DMSO solvent peak. Fig. S9 indicates that DMA molecules were almost removed by the heating, whereas the complicated spectrum implies the deterioration of the sample.

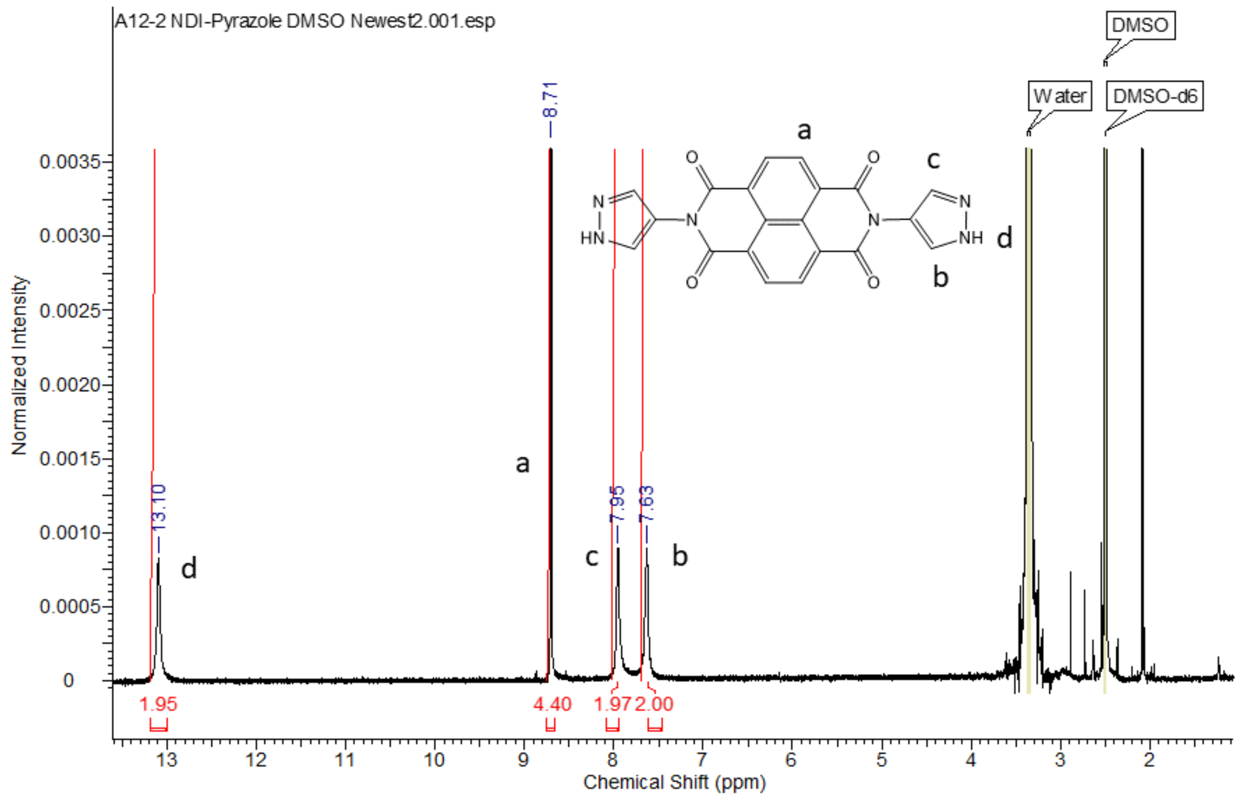


**Fig. S5.** $^{1}$H NMR spectrum of NDI-Hpz dissolved in DMSO-$d_6$ at RT.

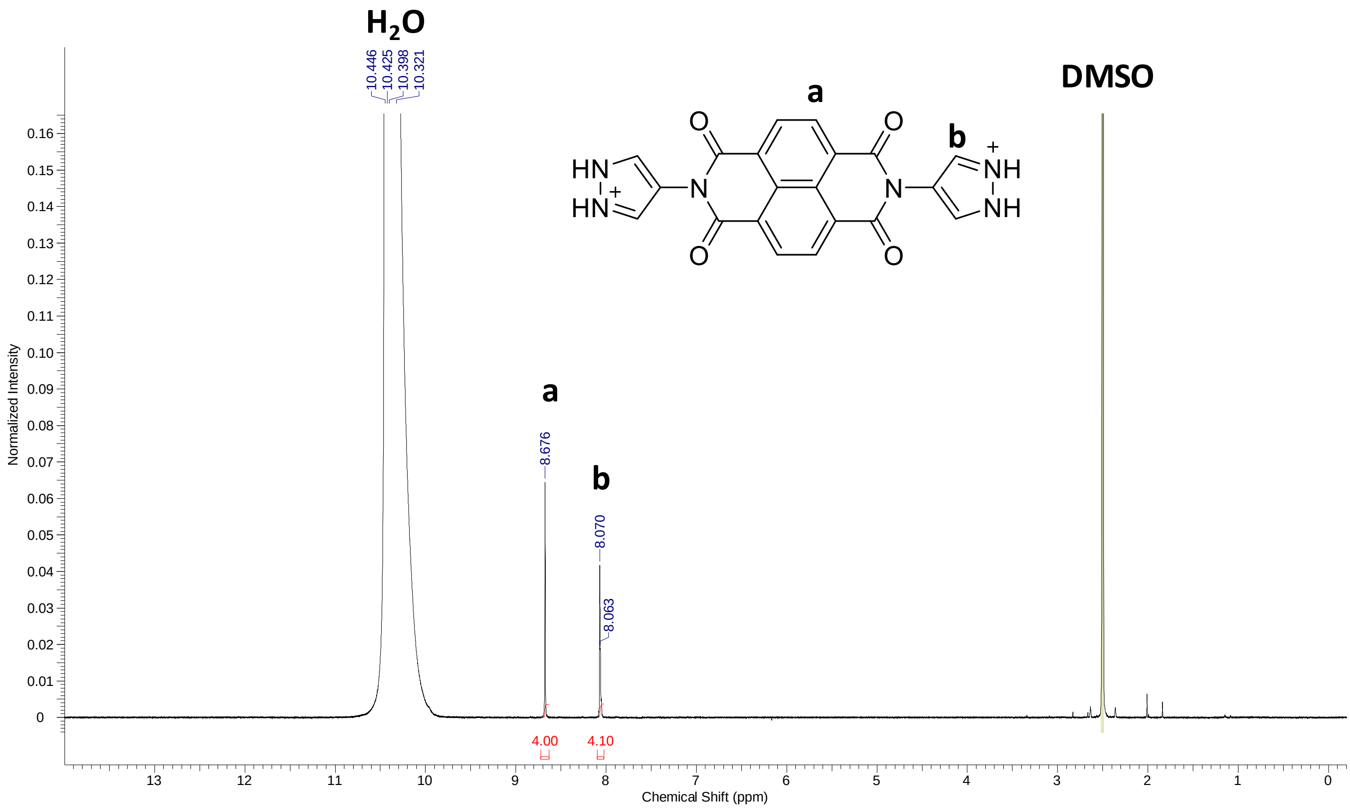


**Fig. S6.** $^{1}$H NMR spectrum of NDI-Hpz dissolved in DMSO-$d_6$ with a drop of conc. $H_2SO_4$ at RT.

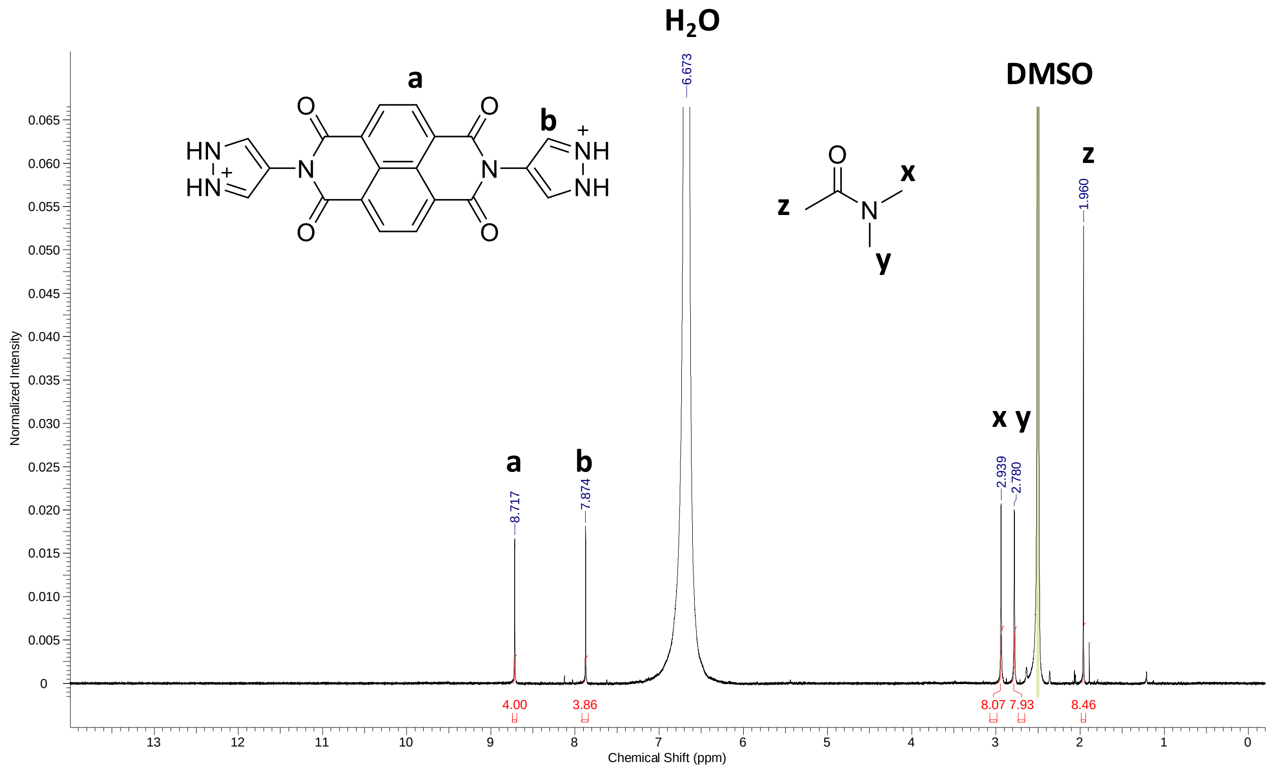


**Fig. S7.** $^{1}$H NMR spectrum of pristine **PMC-Hexagon** dissolved in DMSO-$d_6$ with a drop of conc. $H_2SO_4$ at RT.

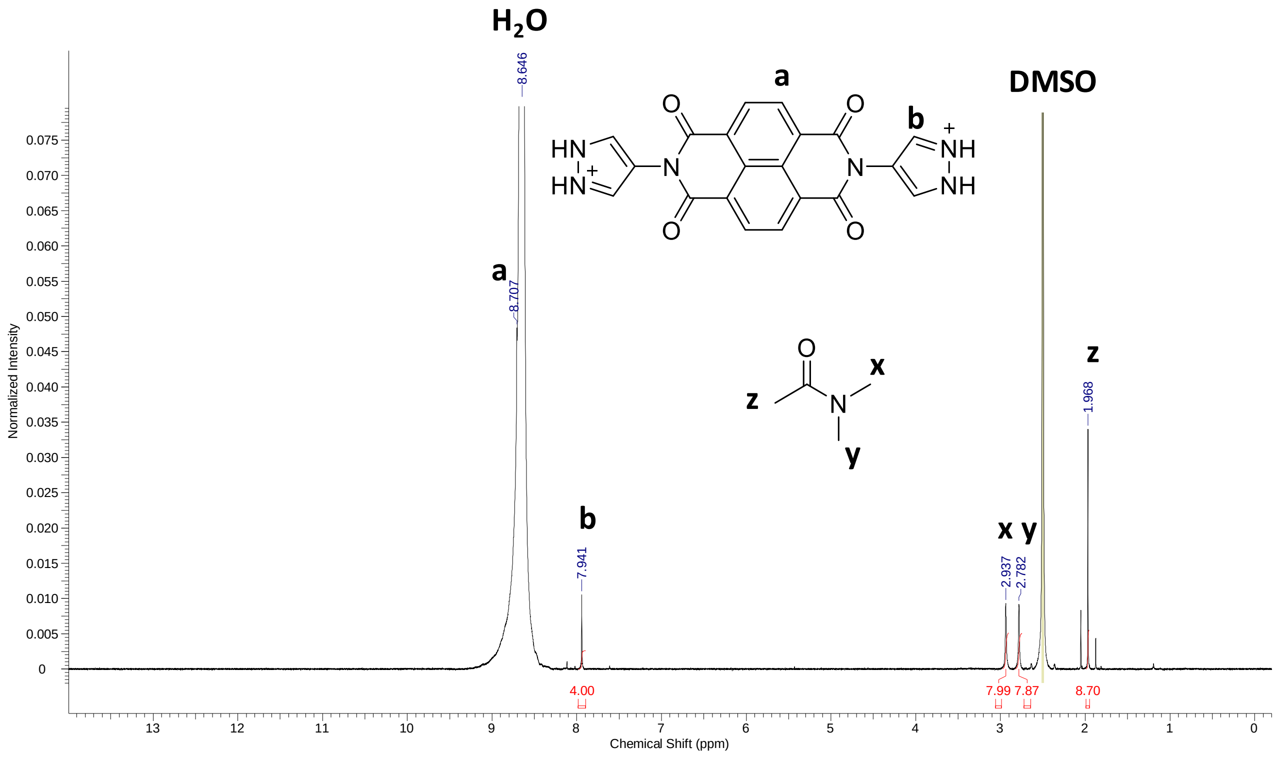


**Fig. S8.** $^{1}$H NMR spectrum of **PMC-Hexagon** (heated at 120 °C for an hour) dissolved in DMSO-$d_6$ with a drop of conc. $H_2SO_4$ at RT.

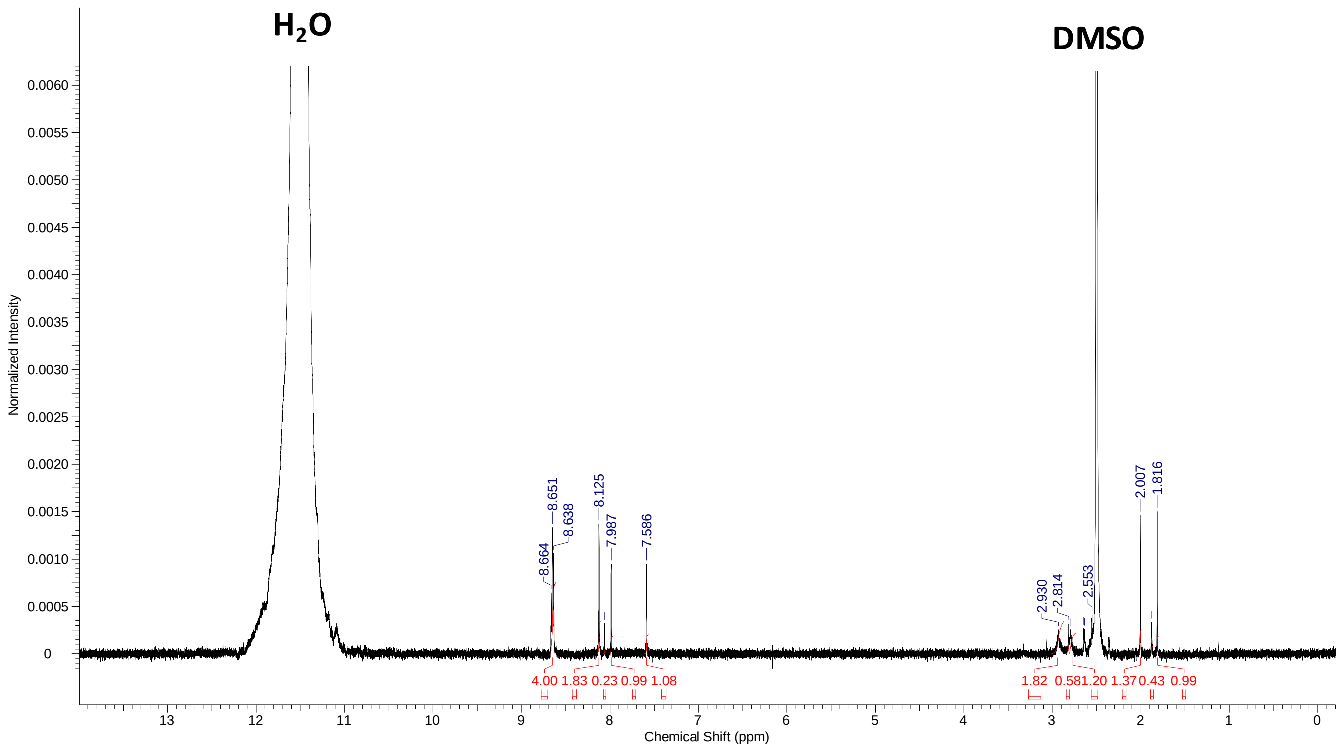


**Fig. S9.** $^{1}$H NMR spectrum of **PMC-Hexagon** (heated at 165 °C for an hour) dissolved in DMSO-$d_6$ with a drop of conc. $H_2SO_4$ at RT.

## $N_2$ sorption isotherm of PMC-hexagon

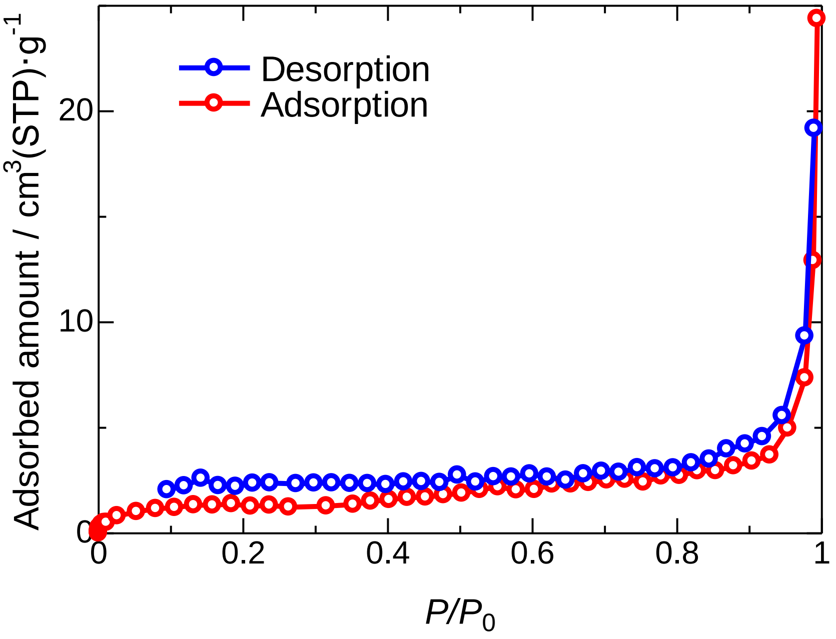


**Fig. S10.** $N_2$ sorption isotherms of **PMC-Hexagon** at 77 K. Red and blue circles represent adsorption and desorption data, respectively.

## PXRD patterns of PMC-hexagon after the measurement of $N_2$ sorption isotherm

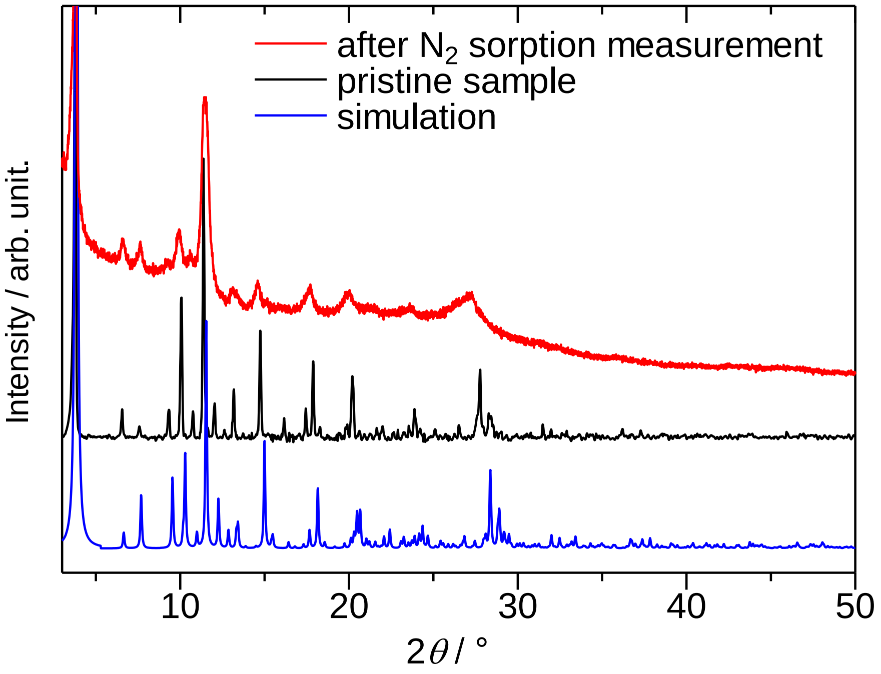


**Fig. S11.** Powder X-ray diffraction (PXRD) patterns of **PMC-hexagon** (simulated from the crystal structure: blue, pristine sample: black, after the measurement of $N_2$ sorption isotherm: red)..

## $^1$H NMR spectrum of PMC-hexagon after the measurement of $N_2$ sorption isotherm

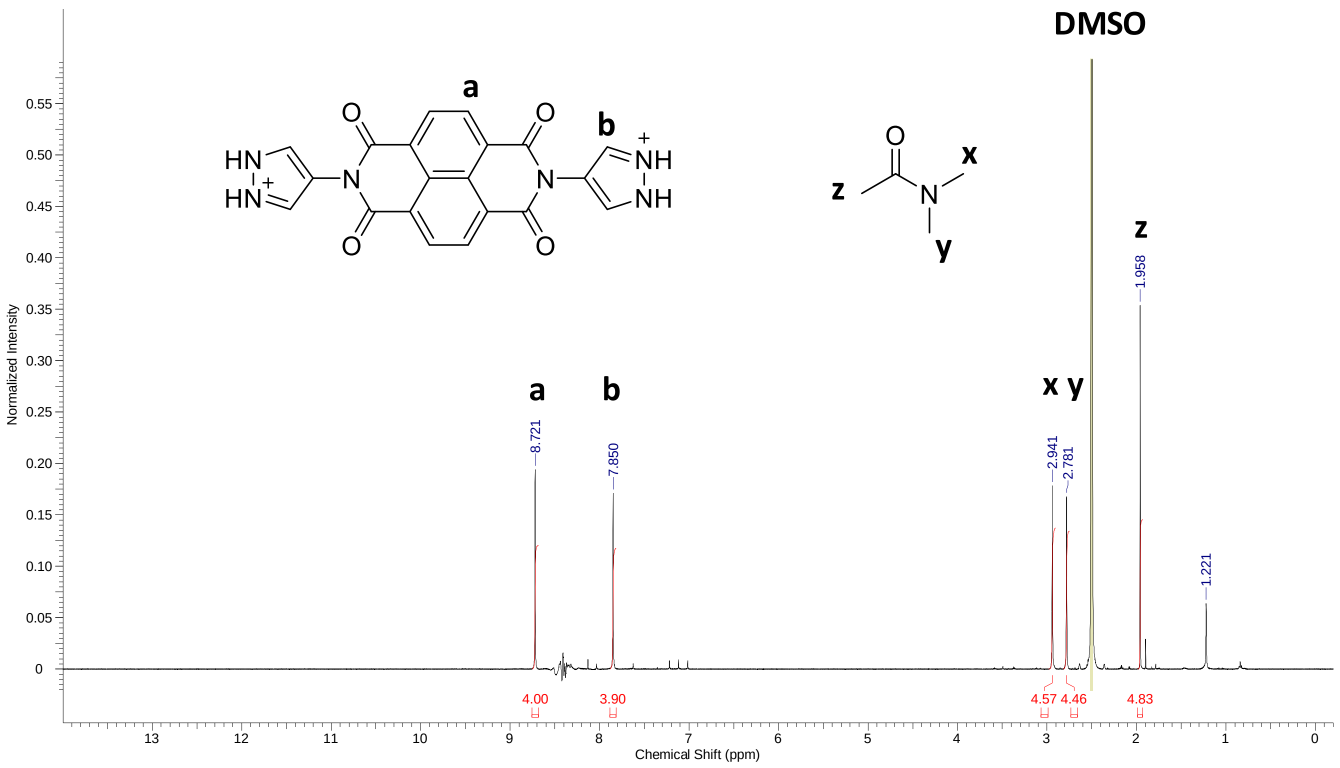


**Fig. S12.** $^1$H NMR spectrum of **PMC-Hexagon** after the measurement of $N_2$ sorption isotherm. The solid was dissolved in DMSO-$d_6$ with a drop of conc. $H_2SO_4$ at RT. Note that strong $H_2O$ signal, which was overlapped with NDI-pyz signals, was carefully removed at the baseline correction process in order to obtain reliable integration values of hydrogens from NDI-pyz ligand. Compared with Fig. S7 and S8, the integration values of **x**, **y** and **z** are nearly half, indicating the elimination of DMA molecules by the activation process.

## References


1 G. A. Bain and J. F. Berry, *J. Chem. Educ.,* 2008, **85**, 532–536.

2 M. C. Burla, R. Caliandro, B. Carrozzini, G. L. Cascarano, C. Cuocci, C. Giacovazzo, M. Mallamo, A. Mazzone and G. Polidori, *J. Appl. Cryst.,* 2015, **48**, 306–309.

3 G. M. Sheldrick, *Acta Crystallogr. Sect. C Struct. Chem.,* 2015, **71**, 3–8.

4 K. Wakita, Yadokari-XG, Software for Crystal Structure Analyses; 2001; Release of Software (Yadokari-XG 2009) for Crystal Structure Analyses, C. Kabuto, S. Akine, T. Nemoto and E. Kwon, *J. Crystallogr. Soc. Jpn.,* 2009, **51**, 218–224.

5 A. L. Spek, *Acta Crystallogr. Sect. C Struct. Chem.,* 2015, **71**, 9–18.

6 G. Velde, F. M. Bickelhaupt, E. J. Baerends, C. F. Guerra, S. J. A. van Gisbergen, J. G. Snijders and T. Ziegler, *J. Comput. Chem.,* 2001, **22**, 931–967.

7 *ADF 2019.3, SCM, Theoretical Chemistry*; Vrije Universiteit: Amsterdam, The Netherlands. Available online: https://www.scm.com/product/adf/.

8 S. Grimme, J. Antony, S. Ehrlich and H. Krieg, *J. Chem. Phys.,* 2010, **132**, 154104.

9 F. Neese, *Wiley Interdiscip. Rev.: Comput. Mol. Sci.,* 2012, **2**, 73–78.

10 F. Neese, *Wiley Interdiscip. Rev.: Comput. Mol. Sci.,* 2018, **8**, 4–9.

11 A. Kubica, J. Kowalewski, D. Kruk and M. Odelius, *J. Chem. Phys.,* 2013, **138**, 064304.
